# Molekularpathologische Untersuchungen im Wandel der Zeit

**DOI:** 10.1007/s00292-024-01326-5

**Published:** 2024-04-15

**Authors:** Maria Walker, Eva-Maria Mayr, Mai-Lan Koppermann, Ana Terron, Yoko Wagner, Charlotte Kling, Nicole Pfarr

**Affiliations:** 1 Institut für Pathologie, Technische Universität München, Trogerstr. 18, 81675 München, Deutschland; 2 Deutsches Konsortium für Translationale Krebsforschung (DKTK), Heidelberg, Deutschland

**Keywords:** DNA, RNA, Exome sequencing, Next Generation Sequencing, Biomarker, DNA, RNA, Exome sequencing, Next generation sequencing, Biomarker

## Abstract

**Hintergrund:**

Molekularpathologische Untersuchungen von Tumorproben umfassen ein weites Spektrum an diagnostischen Analysen. Besonders in den letzten Jahren rückten eine Vielzahl neuer Biomarker in den Vordergrund, deren Analyse für Therapieentscheidungen von großer Bedeutung sind.

**Fragestellung:**

Innerhalb der Molekularpathologie haben die NGS-basierten (Next Generation Sequencing) Anforderungen in den vergangenen Jahren einen massiven Zuwachs erfahren. Um diesen Bedarf abzudecken, werden molekularpathologische Methoden stetig angepasst und weiterentwickelt. Wie dieser Trend zustande kommt und welche Analysen an Bedeutung gewinnen, soll in den folgenden Abschnitten beleuchtet werden.

**Material und Methode:**

Der Artikel gibt einen Überblick der wesentlichen Techniken Nukleinsäure-basierter Analysen aus dem Bereich der massiven Parallelsequenzierung. Es wird in die Terminologie der DNA- und RNA-basierten Diagnoseverfahren sowie die zugehörigen Analysemethoden eingeführt. Der Fokus liegt hierbei auf deren Einsatz in der molekularpathologischen Routinediagnostik.

**Ergebnisse:**

Die Breite der genomischen Sequenzierung nimmt in den letzten Jahren stetig zu, was insbesondere dem Ausbau im Bereich der personalisierten Medizin sowie den steigenden Neuzulassungen von zielgerichteten Therapeutika geschuldet ist. Dadurch wird u. a. die Analyse neuer Biomarker erforderlich. Die Diagnostik im Rahmen eines interdisziplinären molekularen Tumorboards (MTB) erfordert mittlerweile den Einsatz von großen Genpanels (> 1 Megabase). Darüber hinaus wurde durch das Modellvorhaben Genomsequenzierung § 64e erstmalig ein Gesetz erlassen, welches für (austherapierte) onkologische Patienten eine Ganzexom- bzw. Ganzgenomsequenzierung vorsieht. In Hinblick auf diese Entwicklungen liegt es nahe, dass zukünftige Analysen die Integration weiterer Omics-Felder wie der Ganztranskriptomanalyse, des Epigenoms sowie des Proteoms erfordern.

**Schlussfolgerung:**

Die Herausforderungen der personalisierten Medizin sowie die Notwendigkeit des Nachweises einer Vielzahl neuer Biomarker setzen die Implementierung und Umsetzung neuer Techniken in der Molekularpathologie voraus, die eine immer höhere Komplexität aufweisen.

## Abstract (en)

**Background:**

Molecular pathological examinations of tumor samples encompass a wide range of diagnostic analyses. Especially in recent years, numerous new biomarkers have come to the forefront—the analysis of which is crucial for therapy decisions.

**Objectives:**

Within the field of molecular pathology, the demands of next generation sequencing (NGS)-based requirements have experienced massive growth in recent years. To meet this demand, methods are constantly being adapted and further developed. The following sections aim to illuminate how this trend arises and which analyses are gaining importance.

**Methods:**

The article provides an overview of the essential nucleic acid-based analysis techniques in the field of massive parallel sequencing. Terms such as DNA- and RNA-based techniques, as well as the associated analysis methods, are described, particularly with regard to their use in routine molecular pathological diagnostics.

**Results:**

The breadth of genomic sequencing has been steadily growing in recent years, particularly due to the increasing relevance of personalized medicine, along with the rising approvals of targeted therapeutics. This necessitates, among other things, the analysis of new biomarkers. The diagnostics as part of interdisciplinary molecular tumor boards (MTB) are now based on large gene panels (> 1 megabase). Furthermore, through the “Modellvorhaben Genomsequenzierung” § 64e, whole exome or whole genome sequencing has been made available for oncological patients. Given these developments, it is evident that future analyses will require the integration of additional omics fields, such as whole transcriptome analysis, epigenomics, and proteomics.

**Conclusion:**

The challenges of personalized medicine along with the necessity of simultaneously assessing numerous new biomarkers require the implementation and execution of new techniques in molecular pathology whose complexity is steadily increasing.

Die Molekularpathologie beschäftigt sich mit der Analyse pathogener Veränderungen auf der Ebene der Nukleinsäuren (DNA, RNA) zu diagnostischen Zwecken. Das Anforderungsspektrum der molekularpathologischen Untersuchungen umfasst u. a. Erregerdiagnostik, Fragmentlängenanalysen (z. B. Mikrosatelliteninstabilität, Klonalitätsanalysen), klassische PCR-basierte („polymerase chain reaction“) Techniken wie z. B. mutationsspezifische (allelspezifische) PCR zum direkten Nachweis von Punktmutationen in Einzelgenen (z. B. *BRAF*-V600E-Mutationsnachweis), Schmelzkurvenanalysen, Sangersequenzierungen und vieles mehr.

Neben den hier aufgelisteten „klassischen“ molekularpathologischen Untersuchungen kam es in den letzten Jahren zu einer methodischen Verschiebung in den Bereich der massiven Parallelsequenzierung (MPS), auch bekannt als Next Generation Sequencing (NGS). Dies liegt v. a. an der Vielzahl DNA-basierter Biomarker, die in den letzten Jahren etabliert wurden. Die Untersuchung dieser Biomarker ist für die Therapieentscheidungen, insbesondere im Bereich der personalisierten Medizin, aber auch für eine genauere Diagnosestellung von großer Bedeutung. Gleichzeitig wird die verfügbare Menge an Gewebe für molekularpathologische Untersuchungen aus verschiedenen Gründen immer begrenzter. So werden beispielsweise durch die Änderung chirurgischer Verfahren häufig anstelle von großen Resektionen nur noch kleine Biopsien entnommen. Hinzu kommt eine zunehmende Anzahl von Markern, welche zur Diagnosestellung gefordert sind und an diesem begrenzten Material, z. B. durch immunhistochemische Färbungen, getestet werden müssen.

## Qualitätskriterien

Um auch an kleinen Gewebeproben zuverlässige und reproduzierbare Ergebnisse zu gewährleisten ist eine strenge Qualitätskontrolle in jedem Schritt des Arbeitsablaufs erforderlich. Eine nicht ordnungsgemäße Prüfung oder Behandlung des Ausgangsmaterials kann einen negativen Einfluss auf die Qualität und Regelmäßigkeit der Testergebnisse haben. Eine Standardisierung der präanalytischen Variablen kann hilfreich sein, ist aber aufgrund der unterschiedlichen Vorgehensweisen bei Probengewinnung, Probenfixierung und Probenzuschnitt oft nur eingeschränkt umsetzbar [4, 8, 29].

Die Etablierung standardisierter Qualitätskriterien wird dadurch erschwert, dass die Gewebeaufarbeitung zur Extraktion von Nukleinsäure (DNA, RNA oder TNA), die Herstellung der Sequenzierbibliothek sowie die Sequenziertechnik und -plattform auf unterschiedliche Weise erfolgen können. Außerdem ergeben sich durch den Einsatz diverser Assays unterschiedliche Anforderungen an das Ausgangsmaterial, welche ebenfalls berücksichtigt werden müssen [1, 7]. Probenparameter wie z. B. die DNA/RNA-Konzentration, Reinheit, Größenverteilung und Integrität können hierbei zur Beurteilung der Qualität herangezogen werden.

Zusammenfassend erhöht die Komplexität molekularpathologischer Assays das Potenzial von Analysefehlern. Um diesen entgegenzuwirken, müssen in molekulardiagnostischen Labors strenge Qualitätssicherungssysteme eingesetzt werden [40]. Eine weitere Herausforderung für die Zukunft stellt die Umsetzung der IVD-Regularien (In-vitro-Diagnostika) bezüglich der eingesetzten Assays in der Routinediagnostik dar. Hier gilt es das Problem im Umgang mit der Vielzahl an in früheren Jahren etablierter laboreigens entwickelter Techniken/Methoden zu lösen [18].

## DNA-basierte NGS-Analysen

Genomische Untersuchungen, insbesondere Sequenzierverfahren wie Genpanelsequenzierungen, zählen momentan zu den am häufigsten eingesetzten analytischen DNA-basierten Verfahren in der Molekularpathologie. Sie beinhalten wenige bis viele hundert Gene, die parallel untersucht werden können. Die Mindesteinsatzmenge an DNA für diese Genpanelsequenzierung liegt im Bereich von 10 ng (Amplikon-basiert) bis 50 ng (Hybrid-Capture). Um die Mindesteinsatzmenge zu erreichen, werden für Formalin-fixierte, Paraffin-eingebettete (FFPE-)Proben, optimierte, meist automatisierte DNA-Extraktionsprotokolle verwendet.

Aufgrund der bereits beschriebenen Limitation an verfügbarem Material sind in der Molekularpathologie jedoch in einigen Fällen nur Einsatzmengen von maximal 5 ng verfügbar. Dies führte in klinischen Studien teilweise dazu, dass bis zu 50 % der Fälle hinsichtlich spezifischer therapierelevanter Biomarker nicht untersucht werden konnten [14, 16]. In der molekularpathologischen Routinediagnostik ist eine solche Ausfallrate von Proben nicht akzeptabel. Deshalb werden hier limitierte Einsatzmengen durch angepasste Protokolle oder dem Einsatz von Amplikon-basierten Sequenzierpanels kompensiert. Die Zahl der nicht verwertbaren Proben sinkt somit selbst bei kleinen Biopsien auf deutlich <  10 % in der Routinediagnostik.

Durch die Entwicklung der NGS-Technik, der je nach Größe eingesetzten Genpanels und der hierdurch verbundenen Zunahme an generierten Datenmengen werden standardisierte Richtlinien für die Anwendung, Bewertung und Validierung der NGS-Technik in der Diagnostik notwendig [22]. Die derzeitig publizierten Leitlinien für NGS konzentrieren sich überwiegend auf die gezielte Analyse unterschiedlicher Geninhalte und Genregionen mittels Genpanels. Hierbei handelt es sich häufig um ausgewählte Genpanels, die spezifisch auf bestimmte Tumorentitäten zugeschnitten sind und deren Analysen in den Leitlinien bzw. Empfehlungen zur Tumordiagnostik vordefiniert sind [25]. Die Größe und Zusammensetzungen dieser spezifischen Genpanels variieren daher und sind häufig abhängig vom aktuellen Wissensstand bezüglich der Tumorentität bzw. der Verfügbarkeit zugelassener, zielgerichteter Medikamente für die bekannten genetischen Biomarker.

Die beiden zurzeit am häufigsten eingesetzten Techniken sind die Amplikon-basierten und die Hybrid-Capture-basierten Sequenziertechniken. Jedoch finden auch andere Arten der Herstellung der Sequenzierbibliotheken Anwendungen im Labor. In vielen molekularpathologischen Laboren kamen in der Vergangenheit v. a. Amplikon-basierte Genpanels zum Einsatz, da sie lange Zeit die größte Eignung für die Materialien in den Pathologien, dem FFPE, aufwiesen und leicht zu implementieren sind. Zum einen ist der Bedarf an DNA-Einsatzmenge mittels dieser Sequenziertechnik mit ca. 10–20 ng der Geringste, wodurch auch sehr kleine Biopsien mit geringer Nukleinsäureausbeute immer noch sequenziert werden können. Zum anderen ist sowohl die Herstellung der Sequenzierbibliothek über eine PCR mit spezifischen Primern, anschließender Barcode-Ligierung und Aufreinigung bis hin zur Sequenzierung in 1–2 Arbeitstagen durchführbar. Diese Methodik stellt somit eine recht schnelle Art der Sequenzierung dar, ist jedoch durch die Amplifikation und der Art der Sequenzierung in bestimmten Bereichen fehleranfällig (z. B. bei Mononukleotidwiederholungen). Zusätzlich fehlt bei dieser Technik die Möglichkeit, PCR-Duplikate bzw. Fixierungsartefakte durch die Zugabe sog. molekularer Barcodes („molecular barcodes“ [MB] oder „unique molecular identifiers“ [UMI]) zu identifizieren und aus der Analyse zu entfernen.

Als Alternative zur Amplikon-basierten Herstellung von Sequenzierbibliotheken, findet die Hybrid-Capture-basierte Sequenziertechnologie in den letzten Jahren immer häufiger Anwendung in der molekularpathologischen Diagnostik. Lange Zeit war diese Technik aufgrund der hohen Einsatzmenge von Nukleinsäure nur bedingt nutzbar. Durch die Anpassung des Inputs an die Bedürfnisse der molekularpathologischen Routinediagnostik ist inzwischen eine Einsatzmenge von 50 ng DNA für die Herstellung der Sequenzierbibliothek ausreichend. Zusätzlich fand die Verwendung von UMI bereits vor einigen Jahren standardmäßigen Einzug. Die reduzierte Fehlerrate dieser Methodik stellte einen klaren Vorteil gegenüber der Amplikon-basierten Technik dar. Das Ganzexom („whole exome“) zählt zu den größten Hybrid-Capture-basierten Panels, da auch hier während der Herstellung der Sequenzierbibliothek eine Anreichung der (protein)kodierenden Bereiche mittels Capture-Sonden erfolgt.

Durch die Zulassung von mehr oder weniger entitätsübergreifenden Biomarkern (Beispiel TRK-Inhibitoren [Tyrosinkinase]) und Metabiomarkern wie der Tumormutationslast (TMB; [36]), Mikrosatelliteninstabilität (MSI; [41]) und auch der genomischen Instabilität verursacht durch die homologe Rekombinationsdefizienz (HRD; [37]) geriet der Fokus der Paneldiagnostik in den letzten Jahren mehr und mehr in den Bereich der großen „Pan-Tumor“-Panels mit mehreren hundert Genen sowie einer Sequenzabdeckung von größer einer Megabase.

## RNA-basierte NGS-Analysen

Nicht nur die DNA-basierten Sequenziertechniken gehören zu den Standardtechniken der Molekularpathologie, sondern auch NGS-basierte Fusionsnachweise auf RNA-Ebene sind mittlerweile nicht mehr weg zu denken. Das Paradigma im Fusionsnachweis, dass jeder Fusionstreiber nur einen oder wenige Fusionspartner haben und nur in bestimmten Entitäten vorkommen kann, ist nicht mehr gültig. In den letzten Jahren wurde eine Vielzahl von Pan-Cancer- (Fusions)Biomarkern beschrieben, wie z. B. *NTRK1, NTRK2, NTRK3, NRG1, FGFR1, FGFR2, FGFR3*, die multiple Fusionspartner aufwiesen und Entitäten übergreifend auftraten [9, 34, 38].

Für die Fusionsdetektion sind sowohl Amplikon- als auch Hybrid-Capture-basierte Sequenziertechniken weit verbreitet. Zusätzlich kommt eine weitere Technik, die sog. „anchored multiplex PCR (AMP)“-Methode zum Einsatz, bei der eine Einzelstrangamplifikation durchgeführt wird. Sowohl die Hybrid-Capture-basierten als auch die AMP-basierten Techniken erlauben es, neue Fusionen zu identifizieren, sofern die vollständigen Genregionen der Zielgene abgedeckt sind. Die Amplikon-basierten Techniken hingegen erlauben es in erster Linie nur die vom Panel abgedeckten, bekannten Fusionspartner zu identifizieren bzw. in wenigen Fällen durch Zufall zu finden, sofern der jeweilige Primer für das entsprechende Gen im Panel enthalten ist [20, 28]. Vor dem Einsatz der Assays sollte daher immer der Inhalt der abgedeckten Gene/Bereiche geprüft werden bzw. auch auf die zu untersuchende Tumorentität abgestimmt werden.

Zusätzliche RNA-basierte Techniken, die in die Molekularpathologie Einzug halten könnten, sind RNA-basierte Genexpressionsanalysen mittels Gesamt-RNA-Sequenzierung („bulk RNA sequencing“) oder „spatial transcriptomics“ bzw. Einzelzell-RNA-Sequenzierung („single cell RNA sequencing“). Diese Methoden werden bisher überwiegend im Forschungskontext eingesetzt, stellen aber eine wertvolle Ressource für die Validierung bzw. Überprüfung von genomischen Daten dar. Sie ermöglichen es auf genomischer Ebene identifizierte Varianten funktionell näher einzuordnen. Spatial-transcriptomics/scRNA-Sequenzierungen könnten zusätzlich zum näheren Verständnis der Tumorheterogenität beitragen.

## § 64e SGB V und die Auswirkungen auf die Molekularpathologie

Mit dem Gesundheitsversorgungs-Weiterentwicklungsgesetz (GVWG) vom 11.07.2021 (BGBl. I, 2754) werden die molekularen Analysen zur Diagnose von onkologischen als auch seltenen (human)genetischen Erkrankungen nun weiterentwickelt [3]. Durch den § 64e SGB V wurde hierbei ein „Modellvorhaben zur umfassenden Diagnostik und Therapiefindung mittels Genomsequenzierung sowohl bei seltenen als auch bei onkologischen Erkrankungen“ geschaffen. Die Zielsetzung dieses Modellvorhabens ist „die umfangreiche Genomsequenzierung im Rahmen eines strukturierten klinischen Behandlungsablaufs und die darauf aufbauende Datenzusammenführung von klinischen und genomischen Daten in einer Dateninfrastruktur, die eine Analyse der gewonnenen Daten zur Verbesserung der medizinischen Versorgung erleichtert“ (Auszug aus [11]).

Dadurch ergibt sich für die am Modellvorhaben teilnehmenden Molekularpathologien und Institutionen die Vorgabe, schnellstmöglich die Ganzexom- bzw. Ganzgenomsequenzierung zu etablieren und zu validieren. Da die Richtlinien zur Qualitätssicherung während der Durchführung noch nicht gänzlich geklärt sind, bleiben einige Fragen offen. Diese beziehen sich auf die Art des zu untersuchenden Materials (Frischgewebe oder FFPE), die angestrebte Sequenziertiefe, die Vorselektion der zu analysierenden Gene (virtuelles Panel), welches obligat bestimmt werden muss (Mutationen, InDels, Genkopiezahlveränderungen, Metabiomarker TMB, MSI, HRD usw.), die Qualitätskriterien für eine erfolgreiche Sequenzierung, die Einverständniserklärung und vieles mehr. Eine Ganzexom („whole exome sequencing“, WES)-Pilotstudie an Frischgewebe wurde in den Zentren für Personalisierte Medizin Baden-Württemberg bereits erfolgreich durchgeführt und kürzlich publiziert [23]. Momentan erfolgt eine erweiterte Version des WES-Piloten an FFPE-Proben, im Rahmen des Deutschen Netzwerks für Personalisierte Medizin (DNPM), an dem 21 Pathologien deutschlandweit teilgenommen haben. Das Pilotprojekt befindet sich zum aktuellen Zeitpunkt der Manuskripterstellung im Stadium der Auswertung.

Eine weitere relevante Frage ist, welche Instanz den bei der WES bzw. Ganzgenomsequenzierung („whole genome sequencing“, WGS) notwendigen Keimbahnabgleich durchführt. Einige Krebserkrankungen zeigen in bis zu 10 % der Fälle [30, 42], entweder eine Keimbahnveränderung in etablierten (z. B. *BRCA 1/2, PALB2* u. a.) oder in noch nicht definierten Genen, bei denen ein Zusammenhang mit familiären Krebserkrankungen diskutiert wird. Im Rahmen genomweiter Assoziationsstudien konnten z. B. genetische Prädispositionen durch die Identifizierung von Risikoloci bestimmt werden [5, 17, 24]. Deshalb ist ein Keimbahnabgleich bei der WES bzw. WGS von großer Bedeutung. Dieser wird an einigen Standorten durch die Pathologie selbst abgebildet, z. T. jedoch auch in enger Zusammenarbeit mit der jeweiligen Humangenetik.

Die technischen Aspekte der WES ähneln denen einer Sequenzierung mittels eines großen Hybrid-Capture-basierten Genpanels und erfordern einen vergleichbaren Arbeitsaufwand im Labor. Im Falle der WGS ist der Aufwand bei der Sequenzierbibliothekenherstellung sogar geringer, da die Anreicherung der kodierenden Bereiche entfällt. Lange Zeit war der Gedanke, eine WES bzw. WGS Analyse durchzuführen, aufgrund der Limitation der Materialmenge und somit der verfügbaren DNA undenkbar. Jedoch gab es auch hier in den letzten Jahren methodische Weiterentwicklungen, bei denen nun auch mit geringeren Einsatzmengen (10–100 ng) Sequenzierbibliotheken hergestellt werden können. Das bevorzugte Material sowohl für WES als auch für WGS ist jedoch noch immer Frischgewebe. Bestrebungen bestehen aber bereits, die Protokolle für die Verwendung von FFPE-Materialien zu optimieren. Hier gilt es noch herauszufinden, wie hoch die Sequenzabdeckung bei WES/WGS sein sollte, um jeden untersuchten genomischen Bereich mit ausreichender Sequenztiefe abzubilden. Die momentanen Schätzungen liegen bei einer 200-fachen Sequenzabdeckung für WES und einer 100-fachen Abdeckung für WGS sowie einer Abdeckung der Probe für den Keimbahnabgleich von 30- bis 50-fach. Auch hier sollte beachtet werden, dass diese Abdeckung auch entsprechend dem eingesetzten Material, Frischgewebe vs. FFPE, angepasst werden sollte.

Die Ganztranskriptomsequenzierung („whole transcriptome sequencing“, WTS) stellt eine erweiterte RNA-basierte Zusatzuntersuchung zu WES im Rahmen des § 64e dar. Mittels der WES-Technik können Translokationen nur dann detektiert werden, sofern die entsprechenden Bereiche durch die eingesetzten Capture-Proben erfasst werden. Diese Methodik wird bereits im Rahmen von molekular geführten Registerstudien für personalisierte Medizin eingesetzt [15, 31].

Die durch das Modellvorhaben gestellten neuen Herausforderungen an die Molekularpathologie schließen auch die bioinformatische Analyse von WES/WGS-Daten ein. Während bei den fokussierten kleinen Genpanels, die häufig „nur“ Hotspotbereiche therapierelevanter Gene untersuchen, die Analysen relativ schnell und unproblematisch durchführbar sind, da eine Vielzahl der von den Genpanels abgedeckten Varianten bekannte tumor‑/therapierelevante Veränderungen darstellen, gibt es bei der Analyse großer Genpanels (> 1 Mb, 400–600 Gene) bereits die ersten Hindernisse. Hier sind zwar eine Vielzahl tumorrelevanter Gene enthalten, die Kenntnis bezüglich der in diesen Genen identifizierten Varianten und deren Eignung zur Vorhersage von Therapieerfolgen steckt jedoch noch vergleichsweise in den Kinderschuhen. Auch werden im Falle der großen Panels bereits eine große Anzahl von Varianten unklarer Signifikanz identifiziert, zu deren Einfluss auf Tumor bzw. Therapie keinerlei Informationen in der Literatur bzw. den öffentlichen Datenbanken zur Verfügung stehen.

Im Falle von WES/WGS gilt es auch zu klären, welche Gene bei der Mutationsanalyse untersucht werden sollen. Eine vollständige Analyse aller Gene wäre in der im Modellvorhaben angestrebten Laufzeit von ca. 4 Wochen nicht machbar. Daher liegt der initiale Fokus der Mutationsanalysen, ähnlich wie im DKTK-MASTER-Programm (Deutsches Konsortium für Translationale Krebsforschung; [21]) und vielen anderen Studien [26, 32], auf einer Auswahl an ca. 500–1000 tumorassoziierten Genen, einem sog. virtuellen Genpanel. Im Gegensatz dazu sollten Änderungen der Genkopiezahl im kompletten genomischen Bereichs analysiert werden [32]. Zusätzlich sollten Metabiomarker wie TMB, MSI, HRD und auch Mutationssignaturen miterfasst werden [2]. Nur im Falle des vollständigen Fehlens von Veränderungen in den Genen des virtuellen Panels kann es sinnvoll werden, weitere Gene in die Analyse mit aufzunehmen. Aufgrund der fehlenden Kenntnisse über die Relevanz von Genveränderungen außerhalb des bekannten tumorassoziierten Spektrums wäre eine solche Analyse zum momentanen Zeitpunkt jedoch nur rein akademisch interessant.

Auch wenn ein großer Teil der Daten der WGS noch nicht ausreichend interpretiert werden kann, so bietet die Technik dennoch vielversprechende Zukunftsperspektiven. Da sich die bisher durchgeführten Analysen mittels Genpanels bzw. auch WES überwiegend auf die kodierenden Abschnitte der Gene beschränken, werden Promoter und intragenischen Bereiche bisher völlig außer Acht gelassen. Beide Bereiche enthalten Elemente, die Einfluss auf die Expression von Genen nehmen könnten. Beispielsweise ist der Einfluss von sporadisch aktiven transposablen Elementen, wie den Alu-Elementen, auf die genomische Instabilität bereits seit längerer Zeit bekannt [27, 43] und werden mit etwa 0,1 % der menschlichen Erbkrankheiten in Verbindung gebracht [19]. Dies wurde jedoch aufgrund der unzulänglichen Methodik bisher nicht regelhaft untersucht. Eine WGS könnte dies ändern.

Zusammenfassend wird durch die Sammlung der mittels Hochdurchsatzsequenzierungen generierten Daten in den nächsten Jahren ein großer Fundus an neuen, potenziell adressierbaren, molekularen Alterationen entstehen. Diese erlauben den Wechsel vom „Ein-Gen-ein-Medikament-Ansatz“ zu einem auf den jeweiligen Patienten zugeschnitten, umfassenderen multiskalierten Ansatz [40]. Erste Vorarbeiten in dieser Richtung finden bereits in den molekularen Tumorboards statt, auch wenn der Informationsschatz momentan noch durch die reduzierte Anzahl an untersuchten Genregionen eingeschränkt ist.

## Sonstige zukünftige Techniken

Bis auf wenige Ausnahmen, wie *MLH1*- und *MGMT*-Promotormethylierungen, sind in den Molekularpathologien erweiterte epigenetische Untersuchungen eine Rarität. Aktuell werden nur im Bereich der Neuropathologien und der Sarkomdiagnostik Methylierungsarrays zur Diagnosestellung regelhaft eingesetzt. Jedoch könnten diese auch in anderen Bereichen, wie z. B. der Prädiktion des Ansprechens auf bestimmte Therapiestrategien (beispielsweise bzgl. des Ansprechens auf Checkpointblockaden) sehr hilfreich sein [35].

**Figure 1:**
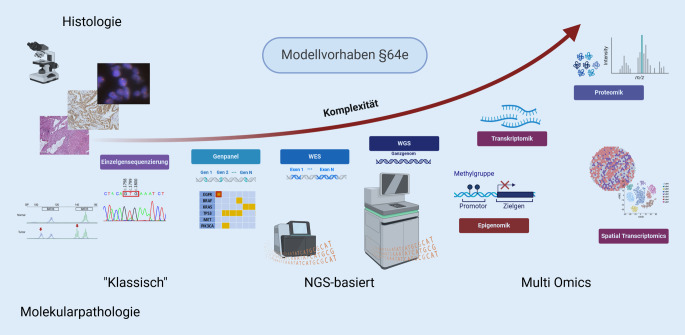

Die Einzelmarkerproteomik in Form von immunhistochemischen Untersuchungen einzelner Biomarker gehört bereits seit vielen Jahren zu dem Standardrepertoire der Pathologie. Der Einsatz von proteomischen (Multiplex‑)Analysen, entweder *in situ* oder aus Extrakten, hat bisher jedoch erst vereinzelt Einzug in die Molekularpathologien und/oder die MTB gehalten. Diese Methodik wäre jedoch gerade in Hinsicht auf die Limitationen des Gewebes (Verfügbarkeit, Tumorzellgehalt) sehr hilfreich, da für multiple Immunhistochemie (IHC) auch multiple Schnitte benötigt werden. Infolgedessen steht in einigen Fällen kein ausreichendes Material mehr für NGS-basierte Analysen zur Verfügung. Das Multiplexen immunbasierter Methoden wie z. B. die Kodetektion durch Indizierung (CODEX; [12]), Zytometrie durch Flugzeitmessung (CyTOF; [13]) aber auch bildgebende Massenspektrometrieverfahren wie „matrix-assisted laser desorption ionization“ (MALDI) sind vielversprechend für die Zukunft der molekularen Diagnostik [33]. Darüber hinaus wird die extraktionsbasierte Flüssigchromatographie mit Massenspektrometriekopplung aus Gewebe für die diagnostische Profilerstellung [10] bereits in einigen wenigen Zentren, wie z. B. dem NCT-MASTER-Programm im Rahmen der personalisierten Medizin, eingesetzt [6, 39]. Die angewandten Technologien sind noch nicht standardisiert, viele Gruppen arbeiten derzeit an einer Verbesserung der Methoden, um diese in die Routinediagnostik einzuführen (Abb. 1).

## Fazit für die Praxis

- Die Molekularpathologie erlebte in den letzten Jahren die Entwicklung einer Vielzahl neuer Biomarker wie Tumormutationslast (TMB) oder homologe Rekombinationsdefizienz (HRD), deren Untersuchung einen Wechsel weg von Einzelgenuntersuchungen hin zu parallelen Hochdurchsatzanalysen erfordert.
- Die NGS-basierten (Next Generation Sequencing) Techniken werden durch das Modellvorhaben § 64e noch weiter in den Fokus rücken. Zusätzlich erfordern der Bereich der personalisierten Medizin und die Suche nach neuen Therapieoptionen für austherapierte Patienten das Fortschreiten der Techniken und deren Einsatz. Hierzu gehört neben WES („whole exome sequencing“) und WGS („whole genome sequencing“) auch der Umschwung auf Multi-Omics-Techniken wie Ganztranskriptomsequenzierung (WTS), Proteomik, epigenetische Untersuchungen etc.
- Die Sammlung dieser Ergebnisse in zugänglichen Datenbanken bzw. Rechenzentren erlaubt darüber hinaus ein neues Spektrum an Möglichkeiten, Forschung durchzuführen um weitere Biomarker identifizieren zu können.
- Die Integration der verschiedenen Omics-Bereiche ermöglicht es in Zukunft, Vorhersagen bzgl. der Ansprechraten von Patienten auf bestimmte Therapien zu treffen.
